# The FCU Online Assessment: A Psychometrically Valid Brief Assessment of Parenting and Child Wellbeing for Parents and Providers

**DOI:** 10.3390/children13060720

**Published:** 2026-05-22

**Authors:** Anna Cecilia McWhirter, Samuel W. Rueter, Jessica N. Tveit, Arin M. Connell, Elizabeth A. Stormshak

**Affiliations:** 1 Northwest Prevention Science, Inc., Eugene, OR 97405, USA; 2 College of Education, University of Oregon, Eugene, OR 97403, USA; srueter@uoregon.edu (S.W.R.); jtveit@uoregon.edu (J.N.T.); bstorm@uoregon.edu (E.A.S.); 3 Department of Psychological Sciences, Case Western Reserve University, Cleveland, OH 44106, USA; amc76@case.edu

**Keywords:** digital parenting interventions, brief assessment, reliability, validity, child wellbeing

## Abstract

**Highlights:**

**What are the main findings?**

- The FCU Online Assessment demonstrated strong reliability among five subscales.
- The FCU Online Assessment demonstrated construct, convergent, and predictive validity.

**What are the implications of the main findings?**

- Parents can use this tool to understand their parenting skills and areas of growth.
- Providers can use this assessment to inform and guide clinical support for parents.

**Abstract:**

**Background/Objectives**: Parenting interventions are an effective way to support child development, and brief screening tools can support equitable implementation of parenting interventions by reducing program costs, increasing accessibility, and engaging populations who have traditionally been underserved. However, brief assessments are frequently overlooked and underutilized. The Family Check-Up (FCU) Online is a digital parenting intervention that integrates a brief FCU Online Assessment, feedback, and parenting skills via an app along with optional provider support. To date, no prior work has validated the FCU Online Assessment. **Method**: The current study combined two samples of parents participating in FCU Online studies and assessed: (1) reliability, (2) construct validity, (3) convergent validity by comparing FCU Online Assessment subscales to similar parenting and child behavior measures, and (4) predictive validity by using FCU Online Assessment at pretest to predict posttest scores as well as parenting and child behaviors at time 2 and time 3. **Results**: Strong reliability was found among all five subscales, including Low Conflict (7 items, *α* = .81), Positive Parenting Practices (11 items, *α* = .80), Positive School Behaviors (5 items, *α* = .83), Consistent Rules and Routines (11 items, *α* = .81), and Child Mental Health (5 items, *α* = .80). The FCU Online Assessment demonstrated construct and convergent validity, as well as predictive validity in that the FCU Online Assessment at pretest predicted posttest scores. **Conclusions**: The FCU Online Assessment is a brief, reliable, and valid measure of parenting and child wellbeing. It can be used by parents and providers alike to evaluate parenting skills and child mental health, develop targeted goals and intervention approaches, and assess family wellbeing over time.

## 1. Introduction

Parenting interventions are an effective way to support child development by improving parents’ family management and communication skills [1]. Though many ‘gold-standard’ parenting interventions (e.g., Triple P, Family Check-Up, Incredible Years) were originally designed for in-person delivery in community health settings, digital and hybrid delivery models have become increasingly popular following the COVID-19 pandemic [2,3]. This digital shift may improve equitable implementation of parenting interventions by reducing program costs, increasing accessibility, and engaging populations who have traditionally been underserved, including rural [4,5,6], low-income [7,8,9], and medically vulnerable families [2,10]. Emerging research suggests that digital parenting interventions may be as effective as in-person interventions [11,12] while improving participant engagement and reducing barriers to participation [13].

A critical component of parenting interventions that is often overlooked is the use of brief assessment screening tools to facilitate parent self-understanding, increase parent motivation, and support providers in engaging parents in digital parenting interventions [14,15,16]. Brief screening tools can provide parents with concrete, actionable, and standardized parenting insight to facilitate self-reflection and intervention engagement [17]. Providers may similarly benefit from utilizing parent response data to guide collaborative conversations with parents completing interventions; assessment tools that are easy to administer and interpret reduce provider burden while supporting efficient intervention delivery [18]. Without such brief assessment tools, provider effort and attention may be misdirected towards supporting parent growth in less pressing parenting domains [19], and parents themselves may struggle to integrate feedback on their parenting skills that does not adequately reflect their strengths or their children’s challenges [20].

Effective integration of reliable and valid measures of parenting and child behavior ensures parenting feedback is more accurate and actionable. This is especially important when feedback is provided to parents in real time, as is the case with meritorious programs like the Family Check-Up Online (FCU Online) [6]. The FCU Online is a digital parenting intervention focused on supporting parents with children aged 18 months–14 years with a range of behavioral and mental health challenges. The program was adapted from the original FCU in-person model [21], which integrates motivational interviewing strategies and emphasizes collaborating with parents to identify child behavioral challenges and implement skills-based parenting strategies over the course of several provider sessions. In the FCU Online, parents complete a built-in brief FCU Online Assessment, receive feedback on assessment results, and access parenting modules within a mobile app. Families additionally have access to optional telehealth provider support as needed. Providers monitor each family’s progress through the app (including results on brief parenting assessment) and contact them to schedule a support session after the completion of each module; families can also contact providers through the app to request support sessions. Telehealth support sessions are brief and typically last only 20–30 min. Provider support includes use of motivational interviewing strategies to increase parent engagement and a skill-building model to inform parent practice and application of skills learned in the app. Research has shown that the FCU Online improves parenting practices (e.g., proactive parenting, limit setting), reduces caregiver stress and mental health challenges, and indirectly improves adolescent behavioral and emotional outcomes by effectuating parenting changes [6,22,23,24]. However, no prior work has evaluated the validity and reliability of the brief FCU Online Assessment integrated into the app itself, which may inform how both parents and their providers engage with the FCU Online as well as how real-time parenting feedback is utilized to support parent, child, and family wellbeing.

The brief FCU Online Assessment covers parenting skills and child behaviors essential to healthy child development. Items cover a range of skills including limit setting, positive and negative communication strategies, quality time, school engagement, healthy routines, and child mental health and behaviors. Parents who manage conflict effectively model healthy problem-solving and communication skills for their children [25]. Consistent articulation and application of family rules and consequences can shape the development of children’s behavior through reinforcement [26], and increasing consistent routines has been linked to decreases in child behavior problems [27]. Parental monitoring of children’s school-based experiences (‘school monitoring’) can facilitate increased connections between home and school, thereby promoting increased academic performance and engagement [28]. Positive parenting practices such as spending quality time with children can increase trust and closeness in parent–child and family relationships [29,30], and positive communication between parents and children can further foster the development of social competence and self-esteem [25,31]. As parenting behaviors have a significant influence on their child’s and adolescents’ mental health and wellbeing, these behaviors are critical to assess. Further, providing parents with timely, accurate feedback on these and similar parenting domains allows them to solidify family management practices that are already working and build necessary skills to meet emergent relational, child behavior, and family climate goals.

Robust empirical support of the FCU and FCU Online has facilitated rapid community-based implementation and uptake efforts across the U.S., as well as internationally [32,33], and an increasing number of community providers are utilizing the FCU Online to support diverse families outside of research-specific service delivery settings. To maximize real-time parenting feedback for parents and providers across varied educational levels and backgrounds, parent progress in the app must be measured in a manner that is easy to understand and captures real parenting experiences. A valid, reliable parenting and child behavior assessment further gives providers useful feedback about the relative effectiveness of their work (e.g., parents not making progress on a parenting skill using only the app may need increased coaching services), thus facilitating accessible, equitable implementation efforts of the FCU Online intervention.

### The Current Study

The current study investigated the reliability and validity of the Family Check-Up (FCU) Online Assessment, a brief screening tool utilized with a sample of parents who previously participated in two research trials using the FCU Online intervention [22,34]. The FCU Online Assessment was used to measure parenting and child behaviors and provide immediate feedback to parents prior to receiving an app-based telehealth behavioral parenting intervention. The current study sought to assess: (1) the reliability of the FCU Online Assessment subscales, (2) construct validity, (3) convergent validity by comparing FCU Online subscales to similar parenting and child behavior measures, and (4) predictive validity by using FCU Online Assessment at pretest scores to predict Assessment posttest scores, as well as parenting and child behaviors at time 2 and time 3. We hypothesized that the FCU Online Assessment would demonstrate adequate reliability (i.e., Cronbach’s internal consistency score of .70 or above) [35], construct validity, and convergent validity using correlational data, as use of correlational analyses is important for establishing measure validity [36]. Finally, we hypothesized that the FCU Online Assessment would demonstrate predictive validity using correlations and general linear model analyses.

## 2. Materials and Methods

### 2.1. Participants and Procedure

Participants originated from two randomized controlled trials investigating the Family Check-Up (FCU) Online intervention, school-age version [22,34], which included parents and caregivers (hereafter solely referred to as parents) of children and early adolescents ages 11–14. Participants in the current study included a subsample of families that completed the FCU Online Assessment (*n* = 243). Table 1 demonstrates family demographic information.

### 2.2. Procedure

The two original randomized controlled trials were conducted in the Pacific Northwest and were funded by the National Institutes of Health (Study 1, Grant R324X220003; Study 2, Grant R01MH122213-01S1); all study protocols were approved by the institutional review board. The sample size for the original studies was determined by the minimum number of families required to have sufficient analytical power.

In Study 1 [34], families were recruited via school-based email communications, which explained the study and included a Qualtrics link where parents could provide their contact information. A research staff member contacted interested parents to explain study details. Eligibility criteria included being a parent or having legal guardianship of children aged 11–14 years experiencing emotional distress and school challenges after the COVID-19 pandemic, fluency in spoken and written English or Spanish, having a smartphone with text messaging capability, and having email access. Interested parents were then mailed a packet with a parent consent form, a youth assent form, and parent and youth questionnaires. Once initial questionnaires and consent/assent forms were returned, families were randomly assigned to two groups, including a treatment condition and no-treatment control group. Treatment included the app plus a family coach. Parents received a login to the FCU Online, where they completed the FCU Online Assessment, received feedback, and were provided with parenting skills online through the app. They were additionally assigned a family coach, who contacted parents via telehealth to help them set up the app, create parenting goals, talk through FCU Online Assessment results, and motivate them to improve parenting practices. Families in both conditions were mailed follow-up questionnaires at 3 months (time 2) and 6 months (time 3). Parents also completed the FCU Online Assessment posttest.

In Study 2 [22], families were recruited using flyers distributed through middle schools, community agencies, pediatric settings, and a statewide mailing to families of middle-school students. Flyers included information about the study and a link to the study website, which asked families to provide contact information to discuss participation. During a screening phone call, research staff provided study details and conducted brief screening measures for study eligibility. Eligibility criteria were the same as Study 1, with the addition of requiring that parents endorse significant depression and/or significant stress associated with the COVID-19 pandemic. Parents were excluded from the study if they did not meet the criteria. Family members who agreed to participate provided active consent, completed an initial questionnaire, and were block-randomized by child gender to ensure balance across conditions. Conditions included a waitlist control versus treatment. All families in treatment were offered the FCU Online with provider coaching support. Parents completed follow-up questionnaires at 2 months (time 2) and 4 months (time 3) after the initial questionnaire. Parents additionally completed the FCU Online Assessment posttest.

### 2.3. Measure

#### 2.3.1. Demographic Survey

Parents completed a demographic questionnaire about themselves and their children. Parent information included their gender, age, race/ethnicity, education level, employment status, and income. Child information included their gender, age, and race/ethnicity.

#### 2.3.2. Family Check-Up (FCU) Online Assessment

The FCU Online Assessment was developed when the Family Check-Up intervention was converted into an online format [6]. It is designed to provide brief, targeted screening of parenting and child behaviors to support the clinical utility of the intervention for both providers and parents. Items were connected to specific intervention modules and used to give parents module-specific feedback to support engagement with app content. Items were derived from prior measures that have been used to evaluate the Family Check-Up, but were simplified, shortened, or adapted to fit our online format and brief assessment. Items were selected based on their ability to predict outcomes. The assessment included 62 items using multiple categorical scales (e.g., 5-point Likert-type scales ranging from 0 [never] to 4 [very often]), as well as continuous numerical variables (e.g., “On a typical day how many servings of fruits and vegetables does your child eat?”). Items covered a range of topics including child mental health (e.g., “My child has been feeling down, depressed, or hopeless”), healthy behaviors and routines (e.g., “I talk to my child positively about body image”; “My child has a regular bedtime routine”), parenting behaviors such as rules, communication strategies, and positive engagement (e.g., “Did you set clear rules that you were willing to enforce?”; “Did you speak calmly with your child when you were upset with them?”; “Did you praise or compliment your child for something done well?”), and school support behaviors (e.g., “Does someone check to see if your child has homework?”).

#### 2.3.3. Child Problem Behaviors (Strengths and Difficulties Questionnaire; SDQ) [38]

Parents reported on their child’s behaviors in the last month on a 3-point Likert-type scale ranging from 0 (not true) to 2 (certainly true). The total score was used (25 items, *α* = .86; e.g., “My child has many worries or often seems worried”).

#### 2.3.4. Parenting Behaviors (Parenting Young Children; PARYC) [39]

Parents reported on parenting behaviors in the past month on a 5-point Likert-type scale ranging from 0 (never) to 4 (very often). Subscale means were calculated and included quality time (5 items, *α* = .74; e.g., “Thinking about parenting your child in the past month, did you spend time with your child in ways that were fun for both of you?”), positive parenting (2 items, *r* = .61; e.g., “Thinking about parenting your child in the past month, did you notice and praise your child’s good behaviors?”), proactive parenting (7 items, *α* = .76; e.g., “Thinking about parenting your child in the past month, did you warn your child before a change of activity was required?”), and limit setting (7 items, *α* = .74; e.g., “Thinking about parenting your child in the past month, did you stick to your rules and not change your mind?”). The PARYC has been validated among families with older children and adolescents [40].

#### 2.3.5. Family Time, Parenting Warmth, and Family Conflict (Community Action for Successful Youth Measure; CASEY) [41]

Parents reported on their relationships and family time and level of warmth with their child on a 5-point Likert-type scale ranging from 1 (not at all) to 5 (very often), and family conflict ranging from 0 (never) to 6 (more than 7 times). Subscale means were calculated and included family time (5 items, *α* = .81; e.g., “Last month, you spent positive time together as a family”), parenting warmth (5 items, *α* = .83; e.g., “This child is open with me about sharing feelings and telling me how things are”), and family conflict (4 items, *α* = .69; e.g., “Last month, my child got his/her way by getting angry”).

#### 2.3.6. School Activity Monitoring [42]

Parents reported on their monitoring of their child’s activities related to school on a 5-point Likert-type scale ranging from 0 (never) to 4 (very often). Mean scores were calculated across items (7 items, *α* = .73; e.g., “How often do you check to see if he/she has homework?”).

#### 2.3.7. Negative Parenting Behaviors (Parenting Scale) [43]

Parents reported on negative parenting behaviors in the past month on a 5-point Likert-type scale ranging from 0 (not at all) to 4 (very often). Mean scores were calculated across items (7 items, *α* = .73; e.g., “In the last month, how often did you criticize your child?”).

### 2.4. Data Analyses

Analyses were conducted with SPSS version 30.0 [44]. Missing data was addressed using pairwise deletions [45], and families without app assessment data were excluded from analyses. Negatively valenced variables in the app data were reverse scored so that higher scores across all variables consistently indicated better behavioral or mental health outcome [46,47]. Six continuous numerical variables were excluded as they were distinct from all other items on a Likert-type scale, which could affect interpretation of analyses [48], leaving a total of 56 items. An exploratory factor analysis (EFA) was conducted using principal axis factoring as the extraction method, as it is more robust due to not assuming normality and supports identifying common variance [47], and a direct Oblimin rotation was used as factors were expected to be correlated. The criteria used to evaluate the number of factors to retain included (a) eigenvalues > 1.0, (b) a minimum of three items loading on any given factor, and (c) examining the scree plot [49]. Reliability analyses were then conducted for each new subscale to test for internal consistency [35]. Construct validity was measured via Pearson correlations among all pretest subscales and between the pretest and posttest for each subscale. Convergent validity was measured with Pearson correlations between the FCU Online Assessment subscales and parent self-reported measures on parenting and child behaviors.

Predictive validity was assessed via multivariate general linear model (GLM) analyses. The first multivariate GLM analysis assessed whether the FCU Online Assessment at pretest predicted posttest responses, controlling for family income as this has been found to impact parenting and child behaviors [50]. The second and third multivariate GLM analyses were conducted to assess whether the FCU Online Assessment predicted parent-reported parenting and child behaviors at time 2 and time 3, respectively, controlling for income and parenting and child behaviors at baseline.

## 3. Results

### 3.1. Exploratory Factor Analysis and Reliability of the FCU Online Assessment

In the EFA, the Kaiser–Meyer–Olkin measure of sampling adequacy was not significant at 0.740, and Bartlett’s Test of Sphericity was statistically significant at *p* < 0.001, indicating we could proceed with interpretation. Results of the EFA demonstrated 17 factors above an eigenvalue of 1. An examination of the screen plot indicated five significant factors; thus, the EFA was re-run to identify items within five factors. The pattern matrix was reviewed, and items were removed that were cross loaded (with a primary loading at least .20 or higher than the secondary loading) [49] or that were loading below .32 (the minimum desired loading of an item) [51]. One final item was removed that impacted factor reliability. Table 2 demonstrates final EFA results. Each of the five factors retained a strong structure and explained 47.58% of the total variance. Reliability analyses were run to determine internal consistency within each of the five factors. Consistent with our hypothesis, internal consistency was strong in each factor (i.e., subscale): Low Conflict (7 items, *α* = .81), Positive Parenting Practices (11 items, *α* = .80), Positive School Behaviors (5 items, *α* = .83), Consistent Rules and Routines (11 items, *α* = .81), and Child Mental Health (5 items, *α* = .80).

### 3.2. Construct Validity

Table 3 demonstrates intercorrelations of FCU Online Assessment subscales among the pretest subscales and between pre- and posttest. Correlations ranged from 0.30 to 0.46, indicating medium correlations. While initial analyses suggested a weak relationship between Positive Parenting Practices and Consistent Rules and Routines (*r* = 0.26, 95% BCa CI [−0.01, 0.53]), bootstrapping (1000 samples) indicated these correlations were not statistically significant. Finally, correlations between pretest and posttest subscales demonstrated medium to strong correlations ranging from 0.36–0.82, all at the *p* < 0.01 level. Consistent with study hypotheses, these results demonstrate evidence of construct validity.

### 3.3. Convergent Validity

Convergent validity was assessed via bootstrapped Pearson correlations between the FCU Online Assessment subscales and similar self-reported measures on parenting and child wellbeing and behaviors. Table 4 demonstrates results of these analyses. Correlations ranged from .15 to .51, indicating small to large correlations, all in the expected direction. These results demonstrate evidence of convergent validity, consistent with study hypotheses.

### 3.4. Predictive Validity

#### 3.4.1. FCU Online Assessment Pretest Predicting Posttest

The first multivariate general linear model (GLM) analysis was conducted to assess whether the FCU Online Assessment at pretest predicted posttest responses, controlling for family income. Results of the regression for Positive Parenting Practices was significant, *F*(1, 65) = 15.25, *p* < 0.001, *η^2^* = .585. Specifically, Positive Parenting Practices at pretest predicted Positive Parenting Practices at posttest, *B* = 0.76, *p* < 0.001, 95% CI [0.57, 0.95]. Results of the regression for Positive School Behaviors was significant, *F*(1, 65) = 16.03, *p* < 0.001, *η^2^* = .598. Specifically, Positive School Behaviors at pretest predicted Positive School Behaviors at posttest, *B* = 0.95, *p* < 0.001, 95% CI [0.73, 1.18].

Results of the regression for Consistent Rules and Routines was significant, *F*(1, 65) = 14.18, *p* < 0.001, *η^2^* = .568. Here, Consistent Rules and Routines at pretest predicted both Consistent Rules and Routines at posttest, *B* = 0.66, *p* < 0.001, 95% CI [0.50, 0.83] and Positive School Behaviors at posttest, *B* = 0.17, *p* = 0.002, 95% CI [0.07, 0.28]. Finally, results of the regression for Child Mental Health were significant, *F*(1, 65) = 5.55, *p* < 0.001, *η^2^* = .340. Specifically, Child Mental Health at pretest predicted Child Mental Health at posttest, *B* = 0.43, *p* < 0.001, 95% CI [0.24, 0.62]. Regression results were not significant for Low Conflict, *F*(1, 65) = 1.92, *p* = 0.106, *η^2^* = .151, or income *F*(1, 65) = 0.867, *p* = 0.509, *η^2^* = .074. Overall, regression results had large effect sizes. These results, along with the high correlations between pre- and posttest scores in Table 3, provide evidence for predictive validity, consistent with study hypotheses.

#### 3.4.2. FCU Online Assessment Predicting Parenting and Child Behaviors at Time 2 and Time 3

Two multivariate GLM analyses were conducted to assess whether the FCU Online Assessment at pretest predicted parent-reported parenting and child behaviors at time 2 and time 3, respectively, controlling for income, parenting, and child behaviors at baseline. For the first analysis predicting behaviors at time 2, regression results for Consistent Rules and Routines were significant, *F*(1, 167) = 2.57, *p* = 0.007, *η^2^* = .154. Specifically, higher levels of Consistent Rules and Routines at pretest predicted less PARYC Positive Parenting at time 2, *B* = −0.29, *p* = 0.021, 95% CI [−0.52, −0.04], and more School Activity Monitoring, *B* = 0.25, *p* = 0.006, 95% CI [0.07, 0.43] at time 2. Regression results were not significant for Low Conflict, *F*(1, 167) = 1.10, *p* = 0.370, *η^2^* = .072, Positive Parenting Practices, *F*(1, 167) = 1.64, *p* = 0.101, *η^2^* = .104, Positive School Behaviors, *F*(1, 167) = 1.20, *p* = 0.297, *η^2^* = .078, or Child Mental Health, *F*(1, 167) = 0.95, *p* = 0.490, *η^2^* = .063. Overall, the regression had a large effect size, and results were somewhat consistent with study hypotheses.

The next analysis focused on predicting behaviors at time 3. Regression results were not significant for any subscales: Low Conflict, *F*(1, 156) = 1.16, *p* = 0.324, *η^2^* = .082, Positive Parenting Practices, *F*(1, 156) = 1.79, *p* = 0.068, *η^2^* = .121, Positive School Behaviors, *F*(1, 156) = 1.56, *p* = 0.125, *η^2^* = .107, Consistent Rules and Routines, *F*(1, 156) = 0.51, *p* = 0.884, *η^2^* = .037, and Child Mental Health, *F*(1, 156) = 1.38, *p* = 0.195, *η^2^* = .096 at time 3. Overall, results of this regression analysis were not consistent with study hypotheses.

## 4. Discussion

The current study evaluated the psychometric properties of the FCU Online Assessment, a brief measure integrated into a digital telehealth program focused on improving parenting skills and supporting child mental and behavioral health. This study used data across two studies examining the FCU Online intervention. A total of *n* = 243 parents with children and early adolescents aged 11–14 completed the FCU Online Assessment, which included items adapted from other standardized measures on parenting and child behaviors. To date, no prior study has evaluated the reliability and validity of the FCU Online Assessment.

The EFA demonstrated five highly reliable subscales of the FCU Online Assessment, including Low Conflict (e.g., low levels of unhealthy conflict such as shouting or someone getting their way by getting angry), Positive Parenting Practices (e.g., positive communication, quality time together), Positive School Behaviors (e.g., few concerns about grades or attendance), Consistent Rules and Routines (e.g., clear rules, routines around bedtime and eating), and Child Mental Health (i.e., few symptoms of anxiety, depression, and stress). These parenting and child behavior subscales are critical to healthy child and early adolescent development, as healthy conflict management promotes effective problem solving and communication [25]. Further, positive parenting practices such as positive communication and quality time can promote social competence and self-esteem [25,31] and can increase closeness and trust in parents’ relationships with their children [29,30], all of which are critical during childhood and the early adolescent period.

Construct validity of the FCU Online Assessment was supported via correlation analyses, demonstrating that subscales were connected to conceptually consistent domains, all in expected directions. For example, Positive School Behaviors was positively correlated with Child Mental Health and with parents using Consistent Rules and Routines, which is consistent with the literature (e.g., [27]). Further, each of the subscales was positively associated between pre- and posttest. Convergent validity was additionally supported, as significant correlations were found between the FCU Online Assessment and measures assessing the same or similar parenting and child behaviors. In particular, the Positive Parenting Practices subscale was significantly associated in the expected direction with all other parenting and child wellbeing measures, and the Consistent Rules and Routines subscale was significantly correlated in the expected direction with most other measures. Finally, all FCU Online Assessment subscales were negatively associated with child problem behaviors and negative parenting behaviors.

Regarding predictive validity, the FCU Online Assessment at pretest significantly predicted posttest scores. Here, most subscales significantly predicted themselves (e.g., Positive Parenting Practices at pretest predicted Positive Parenting Practices at posttest), while Consistent Rules and Routines at pretest additionally predicted Positive School Behaviors at posttest, all with large effect sizes. Correlations between the same subscale and pre- and posttest suggest stability over time, and associations across different subscales over time suggest cross-domain effects. The ability of the brief FCU Online Assessment to predict posttest scores demonstrates that the measure can have clinical significance for both parents and providers in the community. Parents can rely on this measure to provide useful feedback on their parenting skills, and providers can use this brief, efficient measure to support parent progress over time. It was surprising that Low Conflict did not predict any subscales at posttest. As children and early adolescents are consistently developing skills in conflict management, and as forms of conflict between parents and adolescents change during this time of development [52], it is possible that these changes were not completely captured in this brief assessment.

Finally, Consistent Rules and Routines at pretest significantly predicted less positive parenting and more school activity monitoring at time 2, but did not predict other parenting or child behaviors at time 2 or time 3. While inconsistent with hypotheses, it is possible that parent self-report measures for time 2 and time 3 were more related to intervention engagement and the impact of provider coaching support, and therefore these changes would not be sufficiently predicted by pretest app scores. Time 2 and time 3 each also indicated slightly different timepoints across studies (e.g., time 2 for study 1 was 3 months, but for study 2, it was 2 months), which may have also interfered with the predictive validity of the FCU Online Assessment. Notably, parents completed self-report measures as part of participating in a research study; however, parents and providers using the FCU Online in the community will only be relying on the FCU Online Assessment to evaluate parent progress over time. Therefore, the ability of the pretest scores to predict posttest scores will have more value for parents and providers in the community.

### 4.1. Implications

The FCU Online Assessment has sufficient reliability and validity to be used as a brief measure to assess parenting, child behavior, and child mental health, as parents learn parenting skills via this digital parenting intervention. The use of this assessment can allow parents to access critical insight into their skills and support engagement and self-reflection [17], which are important for supporting changes parents want to make in their parenting. Further, the use of this brief, valid, and reliable assessment will be beneficial for providers working with parents. Not only can the measure reduce provider burden due to its ease of administration and interpretation, but providers will be able to use this data to guide their interventions to better support parents [18,19]. Interventions will therefore have the potential to be more targeted, and providers will be able to efficiently evaluate parent progress and outcomes over time.

### 4.2. Limitations and Future Directions

This study is not without limitations. The data is based on parent self-report of parenting and child behaviors, and results may therefore include response bias and reduce generalizability. Racial and ethnic diversity was limited among this sample of parents, and future research should evaluate the reliability and validity of this measure across more racially and ethnically diverse groups. Further, the current study evaluated the use of this measure among parents of children and early adolescents. Future work should investigate the psychometric properties of this measure among other age groups, as well as evaluate possible differences in parenting behaviors based on child age or gender. Finally, fewer parents completed the FCU Online Assessment posttest compared to the pretest. Future work should focus on obtaining more posttest scores to enhance current findings.

## 5. Conclusions

The FCU Online Assessment is a brief, reliable, and valid measure of parenting and child wellbeing. Parents completing this assessment will be able to access easy-to-interpret data on their parenting behaviors that will support their ability to self-reflect on their current skills and engage in the intervention to enhance the growth and development of parenting skills. Providers using this assessment will be able to use this data to guide their intervention strategies with parents and focus on targeting parenting skills requiring the most attention and support. Overall, the FCU Online Assessment has the potential to accurately focus on parenting skills needing attention and allow providers to streamline their interventions to provide efficient, targeted support to parents.

## Figures and Tables

**Table 1:** Parent and Child Demographic Information (*n* = 243).

Parents	*N*	%	*M* (*SD*)
Female	225	92.6	
Mean age (years)			42.6 (6.6)
Race/ethnicity			
White	190	78.2	
Bi/Multiracial	37	15.2	
Hispanic/Latinx	26	10.7	
Black/African American, Asian, Native American, Unknown	16	6.6	
Education			
High school degree/GED or less	27	11.1	
Partial college (≥1 year) or specialized training	39	16.0	
Junior college/Associate’s degree	28	11.5	
College or university graduate (4 years)	78	32.1	
Graduate degree	71	29.2	
Employment status			
Employed full-time	140	57.6	
Employed part-time	35	14.4	
Full-time homemaker	21	8.6	
Self-employed	19	7.8	
Other employment (unemployed, disabled, retired, student, other)	28	11.5	
Income ≤ $49,999 (USD)	87	35.6	
**Children**			
Male	118	48.6	
Female	110	45.3	
Other (e.g., trans, gender fluid, nonbinary)	15	6.1	
Mean age (years)			12.5 (1.0)
Race/ethnicity			
White	172	70.8	
Bi/Multiracial	61	25.1	
Hispanic/Latinx	33	13.6	
Black/African American, Asian, Native American, Unknown	10	4.0	

*M* = Mean; *SD* = Standard deviation. Income: The income cutoff above indicates the number of families in the sample struggling economically. In 2023, the federal poverty line for a family of 4 was approximately $30,000 [37], while 185% of the poverty line for a family of 4 was approximately $55,500 (indicating federal qualification for financial assistance programs).

**Table 2:** Factorial Solution for the Family Check-Up Online Assessment.

Factor and Item	*M* (*SD*)	Factor Loading
**Factor 1: Low Conflict (7 items)**		
1. Problem solving: In the past week, we got angry at each other. ^a^	2.61 (0.81)	.76
2. Problem solving: In the past week, we argued. ^a^	2.57 (0.81)	.69
3. Ask questions: You yell or shout at your child. ^a^	2.67 (0.74)	.67
4. Listening: My child gets angry at me easily.	3.33 (1.15)	.59
5. Discipline: In the past month, did you raise your voice or yell when your child misbehaved? ^a^	2.56 (0.85)	.57
6. Problem solving: In the past week, someone got their way by getting angry. ^a^	3.14 (0.83)	.54
7. Communication: How often is this true? You speak calmly with your child when you were upset with them.	2.77 (0.73)	.45
Eigenvalue Variance Explained		7.33 19%
**Factor 2: Positive Parenting Practices (11 items)**		
1. Encouragement and praise: In the past month, did you praise or compliment your child for something done well?	3.11 (0.78)	.74
2. Encouragement and praise: In the past month, did you notice and praise your child’s good behavior?	3.08 (0.77)	.72
3. Spend quality time: In the past month, did you spend time with your child in ways that were fun for both of you?	2.50 (0.82)	.64
4. Incentives and rewards: In the past month, did you reward your child when they did something well or practiced a new skill?	2.68 (0.83)	.61
5. Spend quality time: In the past month, did you do an enjoyable activity together?	2.43 (0.82)	.57
6. Spend quality time: In the past month, did you help your child learn a new skill?	1.97 (0.83)	.42
7. Giving directions: In the past month, did you tell your child what you would like them to do when they are doing something you don’t like?	2.86 (0.74)	.39
8. Body positivity: I talk to my child positively about body image?	2.61 (0.56)	.39
9. Spend quality time: In the past month, did you spend positive time together as a family?	2.76 (0.84)	.38
10. School routines: How often it typically occurs in your home? Someone checks in with your child about their day.	3.70 (0.53)	.34
11. Giving directions: In the past month, did you tell your child how you expected them to behave (such as at a family gathering)?	2.68 (1.00)	.31
Eigenvalue Variance Explained		3.01 9%
**Factor 3: Positive School Behaviors (5 items)**		
1. School behavior: How concerned are you about the following school behaviors? Gets good grades. ^a^	3.09 (1.06)	.89
2. School behavior: How concerned are you about the following school behaviors? Completes homework and assignments on time. ^a^	2.86 (0.99)	.87
3. School routines: When your child is assigned homework, what percentage of the time do they complete it?	3.09 (1.22)	.66
4. School behavior: How concerned are you about the following school behaviors? Likes going to school. ^a^	3.05 (0.96)	.48
5. School behavior: How concerned are you about the following school behaviors? Shows up on time to school or other activities. ^a^	3.43 (0.90)	.47
Eigenvalue Variance Explained		2.82 7%
**Factor 4: Consistent Rules and Routines (11 items)**		
1. Healthy routines: My child eats meals at the same time every day.	2.88 (0.83)	.65
2. Clear rules/commands: In the past month, did you set clear rules that you were willing to enforce?	3.06 (0.72)	.56
3. Sleep: Does someone make sure your child is in bed on time?	3.07 (0.90)	.54
4. Clear rules/commands: In the past month, did you make sure your child followed the rules that you set?	2.92 (0.68)	.54
5. Healthy routines: My child has a regular bedtime routine.	3.22 (0.87)	.52
6. Clear rules/commands: In the past month, did you stick to your rules and not change your mind?	2.88 (0.73)	.50
7. Healthy routines: My family has a regular morning routine.	3.38 (0.75)	.50
8. Communication: How often is this true? You explain what you wanted your child to do in clear and simple ways.	2.99 (0.70)	.43
9. School routines: How often it typically occurs in your home? Someone checks to see if your child has homework.	2.88 (0.97)	.41
10. Logical consequences: In the past month, did you do something right away when your child misbehaved?	2.84 (0.68)	.39
11. School routines: How often it typically occurs in your home? Someone knows how your child is doing in different subjects at school.	3.37 (0.71)	.36
Eigenvalue Variance Explained		2.52 7%
**Factor 5: Child Mental Health (5 items)**		
1. Emotional well-being: During the past month, how often has your child been bothered by feeling nervous, anxious, or on edge? ^a^	2.96 (0.77)	.82
2. Emotional well-being: During the past month, how often has your child been bothered by not being able to stop or control their worrying? ^a^	3.28 (0.78)	.78
3. Emotional well-being: During the past month, how often has your child been bothered by feeling down, depressed, or hopeless? ^a^	3.37 (0.68)	.72
4. Coping with stress: How would you rate your child’s stress level over the past month? ^a^	1.75 (1.04)	.61
5. Emotional well-being: During the past month, how often has your child been bothered by little interest or pleasure in doing things? ^a^	3.29 (0.76)	.49
Eigenvalue Variance Explained		2.30 6%

*N* = 243. Final solution = 5 factors with 39 total items. Pattern matrix derived with Principal Axis Factoring extraction, direct Oblimin rotation with Kaiser Normalization. ^a^ = Reverse coded prior to EFA; Positive loadings all indicate positive behaviors.

**Table 3:** Pearson Correlations Measuring Construct Validity among Family Check-Up Online Assessment Pre- and Posttest.

	*M*	*SD*	1	2	3	4	5
1. Low Conflict	2.49	0.38	.36 ** [0.18, 0.56]				
2. Positive Parenting Practices	2.74	0.44	.04 [−0.19, 0.25]	.76 ** [0.64, 0.85]			
3. Positive School Behaviors	3.08	0.81	.21 [−0.03, 0.46]	.18 [−0.01, 0.38]	.82 ** [0.71, 0.91]		
4. Consistent Rules and Routines	2.97	0.45	.14 [−0.09, 0.36]	.26 ^a^ [−0.01, 0.53]	.39 ** [0.18, 0.58]	.77 ** [0.66, 0.86]	
5. Child Mental Health	2.79	0.64	.01 [−0.26, 0.33]	−.01 [−0.32, 0.31]	.46 ** [0.21, 0.66]	.30 * [0.08, 0.50]	.57 ** [0.40, 0.72]

*Note. N* = 65. * *p* < 0.05, ** *p* < 0.01. *M* = Mean, *SD* = Standard Deviation. [95% CI] = Bias-Corrected and Accelerated (BCa) Confidence Intervals based on 1000 bootstrap samples. Numbers below the diagonal indicate correlations between pretest subscales. Bolded numbers along the diagonal indicate correlations between pre- and posttest subscales. ^a^ = BCa CI indicates value not significant after bootstrapping; original value appeared significant at the *p* < 0.05 level.

**Table 4:** Pearson Correlations Evaluating Convergent Validity of Family Check-Up Online Assessment.

	Low Conflict	Positive Parenting Practices	Positive School Behaviors	Consistent Rules and Routines	Child Mental Health
SDQ total problem behaviors	−.31 ** [−0.44, 0.60]	−.17 * [−0.32, −0.02]	−.49 ** [−0.60, −0.38]	−.28 ** [−0.41, −0.14]	−.51 ** [−0.60, −0.41]
PARYC quality time	−.63 [−0.22, 0.09]	.40 ** [0.25, 0.53]	.41 [−0.09, 0.16]	.23 ** [0.08, 0.37]	.09 [−0.06, 0.24]
PARYC positive parenting	−.09 [−0.23, 0.06]	.44 ** [0.31, 0.56]	−.06 [−0.20, 0.06]	.09 [−0.06, 0.23]	.04 [−0.11, 0.20]
PARYC proactive parenting	−.05 [−0.21, 0.12]	.31 ** [0.16, 0.45]	−.15 * [−0.28, −0.03]	.14 [−0.02, 0.29]	−.07 [−0.22, 0.11]
PARYC limit setting	−.03 [−0.18, 0.12]	.25 ** [0.10, 0.38]	−.06 [−0.20, 0.07]	.22 ** [0.07, 0.35]	−.05 [−0.19, 0.10]
CASEY Family time	.06 [−0.09, 0.23]	.50 ** [0.38, 0.61]	.12 [−0.03, 0.26]	.22 ** [0.08, 0.36]	.18 * [0.03, 0.32]
CASEY Parenting warmth	−.01 [−0.16, 0.15]	.42 ** [0.31, 0.51]	.09 [−0.04, 0.22]	.29 ** [0.16, 0.41]	.20 ** [0.04, 0.36]
CASEY Family conflict	−.40 ** [−0.52, −0.28]	−.25 ** [−0.38, −0.13]	−.13 [−0.28, 0.02]	−.19 * [−0.33, −0.04]	−.29 ** [−0.41 −0.17]
School activity monitoring	−.05 [−0.21, 0.11]	.22 ** [0.09, 0.35]	.05 [−0.10, 0.20]	.20 ** [0.06, 0.33]	.03 [−0.12, 0.19]
Negative parenting behaviors	−.35 ** [−0.50, −0.21]	−.33 ** [−0.46, −.18]	−.20 ** [−0.33, −0.03]	−.27 ** [−0.41, −0.13]	−.29 ** [−0.44, −0.15]

*N* = 181. * *p* < 0.05, ** *p* < 0.01. SDQ = Strengths and Difficulties Questionnaire; PARYC = Parenting Young Children; CASEY = Community Action for Successful Youth Measure. [95% CI] = Bias-Corrected and Accelerated (BCa) Confidence Intervals based on 1000 bootstrap samples. Bolded numbers are significant.

## Data Availability

Data can be made available upon request to Elizabeth A. Stormshak; the original studies were pre-registered on ClinicalTrials.gov (NCT05400564, NCT03060291) and approved by the institutional review board (IRB).

## Abbreviations

The following abbreviations are used in this manuscript:

FCU	Family Check-Up
EFA	Exploratory Factor Analysis
GLM	General Linear Model
SDQ	Strengths and Difficulties Questionnaire
PARYC	Parenting Young Children
CASEY	Community Action for Successful Youth Measure
